# Avian Influenza A (H7N9) Model Based on Poultry Transport Network in China

**DOI:** 10.1155/2018/7383170

**Published:** 2018-11-04

**Authors:** Juping Zhang, Wenjun Jing, Wenyi Zhang, Zhen Jin

**Affiliations:** ^1^Complex Systems Research Center, Shanxi University, Taiyuan, Shanxi 030006, China; ^2^Shanxi Key Laboratory of Mathematical Techniques and Big Data Analysis on Disease Control and Prevention, Shanxi University, Taiyuan, Shanxi 030006, China; ^3^Institute of Disease Control and Prevention of PLA, Beijing 100071, China

## Abstract

In order to analyze the spread of avian influenza A (H7N9), we construct an avian influenza transmission model from poultry (including poultry farm, backyard poultry farm, live-poultry wholesale market, and wet market) to human according to poultry transport network. We obtain the threshold value for the prevalence of avian influenza A (H7N9) and also give the existence and number of the boundary equilibria and endemic equilibria in different conditions. We can see that poultry transport network plays an important role in controlling avian influenza A (H7N9). Finally, numerical simulations are presented to illustrate the effects of poultry in different places on avian influenza. In order to reduce human infections in China, our results suggest that closing the retail live-poultry market or preventing the poultry of backyard poultry farm into the live-poultry market is feasible in a suitable condition.

## 1. Introduction

**Figure 1:**
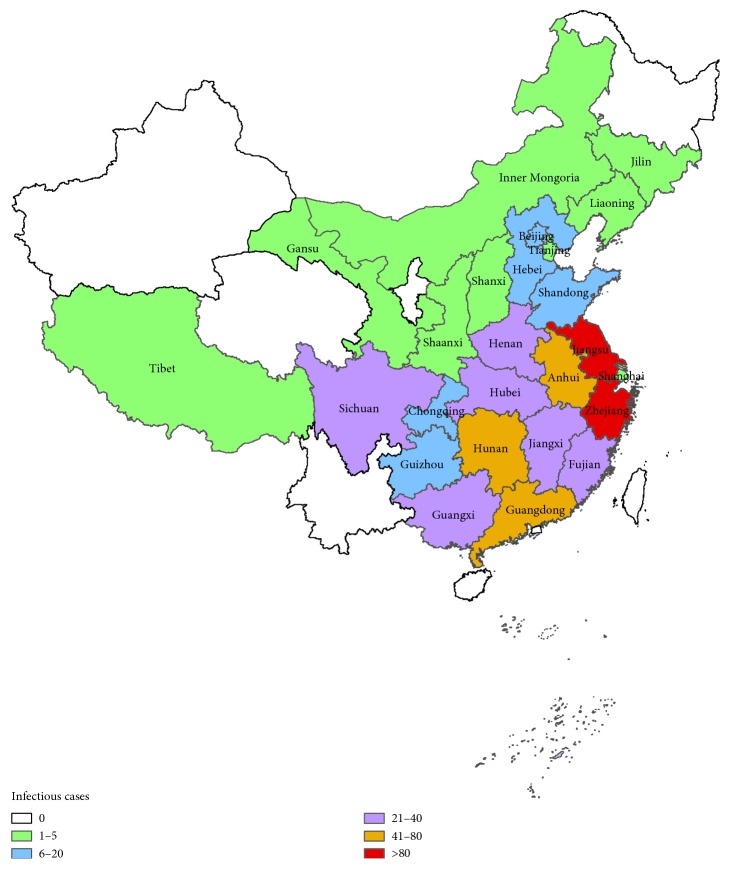
Provinces with avion influenza A (H7N9) from September 2016 to May 2017.

Avian influenza A (H7N9) is a subtype of influenza viruses that have been detected in birds in the past. Until 2013 outbreak in China, no human infections with H7N9 viruses had ever been reported. But from March 31 to August 31, 2013, 134 cases had been reported in mainland China, resulting in 45 deaths [1]. However, the virus came back in November 2013 again. Afterwards the disease came back in November every year. In fact, the second outbreak occurred from November 2013 to May 2014. The third outbreak occurred from November 2014 to June 2015. The fourth outbreak occurred from November 2015 to June 2016. And the fifth outbreak occurred from September 2016 to May 2017 (NHFPC [1]). The disease causes a high death rate. In China, from March 2013 to May 2017, H7N9 has resulted in 1263 human cases including 459 deaths with a death rate of nearly 37%. In China, from September 2016 to May 2017, provinces with human cases are shown as Figure 1. H7N9 virus does not induce clinical signs in poultry and is classified as a low pathogenicity avian influenza virus (LPAIV) [2]. However, the virus can infect humans and most of the reported cases of human H7N9 infection have resulted in severe respiratory illness [3].

Jones et al. [4] demonstrated that interspecies transmission of H7N9 virus occurs readily between society finches and bobwhite quail but only sporadically between finches and chickens, and transmission occurs through shared water. Pantin-Jackwood et al. [3] showed that quail and chickens are susceptible to infection, shed large amounts of virus, and are likely important in the spread of the virus to humans, and it is therefore conceivable that passerine birds may serve as vectors for transmission of H7N9 virus to domestic poultry [4]. Zhang et al. [5] concluded that migrant birds are the original infection source. Many authors investigated the epidemic model which describes the transmission of avian influenza among birds and humans [8–15]. Liu et al. [16] constructed two avian influenza bird-to-human transmission models with different growth laws of the avian population, one with logistic growth and the other with Allee effect, and analyzed their dynamical behavior. Lin et al. [17] developed three different SIRS models to fit the observed human cases between March 2013 and July 2015 in China and found that environmental transmission via viral shedding of infected chickens had contributed to the spread of H7N9 human cases in China. Chen and Wen [18] took into account gene mutation in poultry. Guo et al. [19] proposed and analyzed an SE-SEIS avian-human influenza model. Mu and Yang [20] analyzed an SI-SEIR avian-human influenza model with latent period and nonlinear recovery rate. Gourley et al. [21] analyzed the patchy model for the spatiotemporal distribution of a migratory bird species. Bourouiba et al. [22] investigated the role of migratory birds in the spread of H5N1 avian influenza among birds by considering a system of delay differential equations for the numbers of birds on patches, where the delays represent the flight times between patches. In China, in 2013, to control the outbreak, local authorities of the provinces and municipalities, such as Jiangsu, Shanghai, and Zhejiang, temporarily closed the retail live-poultry markets which proved to be an effective control measure. Data indicate that the novel avian influenza A (H7N9) virus was most likely transmitted from the secondary wholesale market to the retail live-poultry market and then to humans [6, 7]. How is avian influenza A (H7N9) transmitted from live-poultry to human in China? In order to reveal the fact, the global network model of avian influenza A (H7N9) is constructed based on poultry transport network. The relationship between the global system and subsystem is analyzed. The corresponding risk indices are obtained. We study the impact of subsystems on the risk index of the global system. When the disease occurs, it can provide theoretical guidance for the global and local transport of poultry.

In this paper, we construct an avian influenza A (H7N9) transmission model from live poultry (including poultry farm, backyard poultry farm, live-poultry wholesale market, and wet market) to human for the heterogenous environments which affect the spread of H7N9. The remaining part of this paper is organized as follows: in Section 2, we first establish the model based on poultry transport network. We derive the threshold value of the model. In Sections 3 and 4, we discuss the different boundary and endemic equilibrium in the different thresholds.  Section 5 gives the effect of different transmission rate on H7N9 by numerical simulation. Finally, concluding remarks are made in Section 6.

## 2. Model Based on Poultry Transport Network

**Figure 2:**
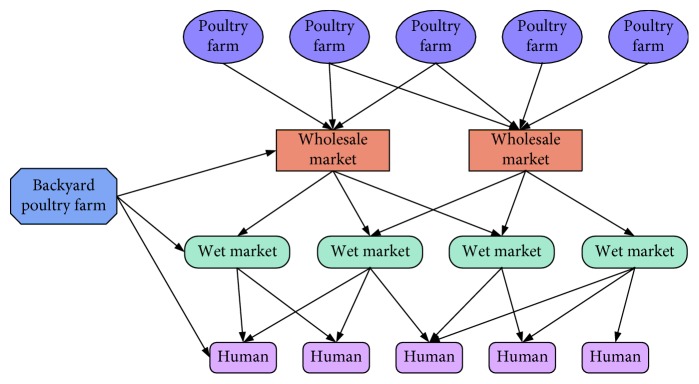
A possible network of H7N9 avian influenza.

**Figure 3:**
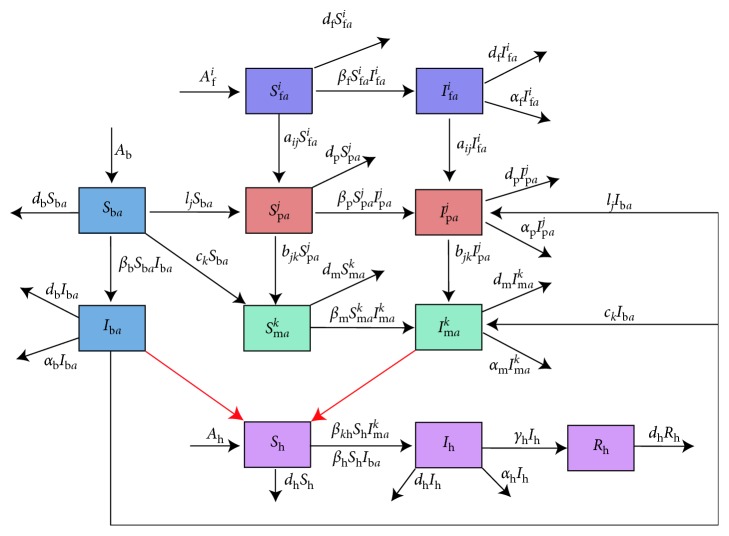
Detailed transfer diagram on the dynamical transmission of H7N9 avian influenza.

The avian population is classified into poultry farm, backyard poultry farm, live-poultry wholesale market, and wet market (the retail live-poultry market). According to the present situation in China, the backyard poultry feeding is regarded as a large node, which is considered to be connected with all other nodes (except poultry farm) in network. The relationship diagram of poultry transport and contacts between human and poultry are described in Figure 2. Let *N*_f*a*_^*i*^(*t*), *N*_p*a*_^*j*^(*t*), and *N*_m*a*_^*k*^(*t*) be the total number of poultry in *i*th poultry farm, *j*th live-poultry wholesale market, and *k*th wet market at time *t*, respectively, where *N*_f*a*_^*i*^(*t*), *N*_p*a*_^*i*^(*t*), and *N*_m*a*_^*i*^(*t*) are classified into two subclasses: susceptible and infective, denoted by *S*_f*a*_^*i*^(*t*) and *I*_f*a*_^*i*^(*t*), *S*_p*a*_^*j*^(*t*) and *I*_p*a*_^*j*^(*t*), and *S*_m*a*_^*k*^(*t*) and *I*_m*a*_^*k*^(*t*), respectively. Suppose there are *L* poultry farms, *M* live-poultry wholesales, and *K* wet markets, namely, *i*=1,…, *L*; *j*=1,…, *M*; *k*=1,…, *K*. And they are independent of each other. Let *N*_h_(*t*) be the total number of human at time *t*. The human population is classified into three subclasses: susceptible, infective, and recovered, denoted by *S*_h_(*t*), *I*_h_(*t*), and *R*_h_(*t*), respectively. All new recruitments of human population and avian population are susceptible. The avian influenza virus is not contagious from an infective human to a susceptible human. It is only contagious from an infective avian to a susceptible avian and a susceptible human. An infected avian keeps in the state of disease and cannot recover, but an infected human can recover, and the recovered human has permanent immunity. We neglect death rates of the poultry individuals during the transport process. The detailed description of dynamical transmission of H7N9 avian influenza is described in the following flowchart (Figure 3).

The corresponding dynamical model can be seen in the following equation:

(1) $$\begin{array}{c} \left\{\begin{array}{c} \frac{dS_{\text{f}a}^{i}\left(t\right)}{dt}=A_{\text{f}}^{i}-\beta_{\text{f}}S_{\text{f}a}^{i}I_{\text{f}a}^{i}-d_{\text{f}}S_{\text{f}a}^{i}-\underset{j}{\sum }a_{ij}S_{\text{f}a}^{i},\\ \frac{dI_{\text{f}a}^{i}\left(t\right)}{dt}=\beta_{\text{f}}S_{\text{f}a}^{i}I_{\text{f}a}^{i}-d_{\text{f}}I_{\text{f}a}^{i}-\alpha_{\text{f}}I_{\text{f}a}^{i}-\underset{j}{\sum }a_{ij}I_{\text{f}a}^{i},\;\;\;i=1,\ldots ,L,\\ \frac{dS_{\text{b}a}\left(t\right)}{dt}=A_{\text{b}}-\beta_{\text{b}}S_{\text{b}a}I_{\text{b}a}-d_{\text{b}}S_{\text{b}a}-\underset{j}{\sum }l_{j}S_{\text{b}a}-\underset{k}{\sum }c_{k}S_{\text{b}a},\\ \frac{dI_{\text{b}a}\left(t\right)}{dt}=\beta_{\text{b}}S_{\text{b}a}I_{\text{b}a}-d_{\text{b}}I_{\text{b}a}-\alpha_{\text{b}}I_{\text{b}a}-\underset{j}{\sum }l_{j}I_{\text{b}a}-\underset{k}{\sum }c_{k}I_{\text{b}a},\\ \frac{dS_{\text{p}a}^{j}\left(t\right)}{dt}=\underset{i}{\sum }a_{ij}S_{\text{f}a}^{i}+l_{j}S_{\text{b}a}-\beta_{\text{p}}S_{\text{p}a}^{j}I_{\text{p}a}^{j}-d_{\text{p}}S_{\text{p}a}^{j}-\underset{k}{\sum }b_{jk}S_{\text{p}a}^{j},\;j=1,\ldots ,M,\\ \frac{dI_{\text{p}a}^{j}\left(t\right)}{dt}=\underset{i}{\sum }a_{ij}I_{\text{f}a}^{i}+l_{j}I_{\text{b}a}+\beta_{\text{p}}S_{\text{p}a}^{j}I_{\text{p}a}^{j}-d_{\text{p}}I_{\text{p}a}^{j}-\alpha_{\text{p}}I_{\text{p}a}^{j}-\underset{k}{\sum }b_{jk}I_{\text{p}a}^{j},\\ \frac{dS_{\text{m}a}^{k}\left(t\right)}{dt}=\underset{j}{\sum }b_{jk}S_{\text{p}a}^{j}+c_{k}S_{\text{b}a}-\beta_{\text{m}}S_{\text{m}a}^{k}I_{\text{m}a}^{k}-d_{\text{m}}S_{\text{m}a}^{k},\\ \frac{dI_{\text{m}a}^{k}\left(t\right)}{dt}=\underset{j}{\sum }b_{jk}I_{\text{p}a}^{j}+c_{k}I_{\text{b}a}+\beta_{\text{m}}S_{\text{m}a}^{k}I_{\text{m}a}^{k}-d_{\text{m}}I_{\text{m}a}^{k}-\alpha_{\text{m}}I_{\text{m}a}^{k},\;k=1,\ldots ,K,\\ \frac{dS_{\text{h}}\left(t\right)}{dt}=A_{\text{h}}-\underset{k}{\sum }\beta_{k\text{h}}S_{\text{h}}I_{\text{m}a}^{k}-\beta_{\text{h}}S_{\text{h}}I_{\text{b}a}-d_{\text{h}}S_{\text{h}},\\ \frac{dI_{\text{h}}\left(t\right)}{dt}=\underset{k}{\sum }\beta_{k\text{h}}S_{\text{h}}I_{\text{m}a}^{k}+\beta_{\text{h}}S_{\text{h}}I_{ba}-d_{\text{h}}I_{\text{h}}-\alpha_{\text{h}}I_{\text{h}}-\gamma_{\text{h}}I_{\text{h}},\\ \frac{dR_{\text{h}}\left(t\right)}{dt}=\gamma_{\text{h}}I_{\text{h}}-d_{\text{h}}R_{\text{h}}. \end{array}\right. \end{array}$$

The interpretations of parameters of system (1) are described in Table 1. The parameters in system (1) are all nonnegative constants.

The variation of the number of poultry in *i*th poultry farm *N*_f*a*_^*i*^(*t*) is

(2) $$\begin{array}{c} \frac{dN_{\text{f}a}^{i}\left(t\right)}{dt}=A_{\text{f}}^{i}-d_{\text{f}}N_{\text{f}a}^{i}-\alpha_{\text{f}}I_{\text{f}a}^{i}-\underset{j}{\sum }a_{ij}N_{\text{f}a}^{i}, \end{array}$$

and thus,

(3) $$\begin{array}{c} N_{\text{f}a}^{i}\left(t\right)\leq \frac{A_{\text{f}}^{i}}{d_{\text{f}}+\sum_{j}a_{ij}}=W_{\text{f}a}^{i}. \end{array}$$

Similarly, the variation of the number of poultry in backyard poultry farm *N*_b*a*_(*t*) is

(4) $$\begin{array}{c} \frac{dN_{\text{b}a}\left(t\right)}{dt}=A_{\text{b}}-d_{\text{b}}N_{\text{b}a}-\alpha_{\text{b}}I_{\text{b}a}-\underset{j}{\sum }l_{j}N_{\text{b}a}-\underset{k}{\sum }c_{k}N_{\text{b}a}, \end{array}$$

and thus,

(5) $$\begin{array}{c} N_{\text{b}a}\left(t\right)\leq \frac{A_{\text{b}}}{d_{\text{b}}+\sum_{j}l_{j}+\sum_{k}c_{k}}=W_{\text{b}a}. \end{array}$$

The variation of the number of poultry in *j*th live-poultry wholesale market *N*_p*a*_^*j*^(*t*) is

(6) $$\begin{array}{c} \frac{dN_{\text{p}a}^{j}\left(t\right)}{dt}=\underset{i}{\sum }a_{ij}N_{\text{f}a}^{i}+l_{j}N_{\text{b}a}-d_{\text{p}}N_{\text{p}a}^{j}-\alpha_{\text{p}}I_{\text{p}a}^{j}-\underset{k}{\sum }b_{jk}N_{\text{p}a}^{j}, \end{array}$$

and thus,

(7) $$\begin{array}{c} N_{\text{p}a}^{j}\left(t\right)\leq \frac{\sum_{i}a_{ij}N_{\text{f}a}^{i}+l_{j}N_{\text{b}a}}{d_{\text{p}}+\sum_{k}b_{jk}}\leq \frac{\sum_{i}\left(\left(\left(a_{ij}A_{\text{f}}^{i}\right)/\left(d_{\text{f}}+\sum_{j}a_{ij}\right)\right)+\left(\left(l_{j}A_{\text{b}}\right)/\left(d_{\text{b}}+\sum_{j}l_{j}+\sum_{k}c_{k}\right)\right)\right)}{d_{\text{p}}+\sum_{k}b_{jk}}=W_{\text{p}a}^{j}. \end{array}$$

The variation of the number of poultry in *k*th wet market *N*_m*a*_^*k*^(*t*) is

(8) $$\begin{array}{c} \frac{dN_{\text{m}a}^{k}\left(t\right)}{dt}=\underset{j}{\sum }b_{jk}N_{\text{p}a}^{j}+c_{k}N_{\text{b}a}-d_{\text{m}}N_{\text{m}a}^{k}-\alpha_{\text{m}}I_{\text{m}a}^{k}, \end{array}$$

and thus

(9) $$\begin{array}{c} N_{\text{m}a}^{k}\left(t\right)\leq \frac{\sum_{j}b_{jk}N_{\text{p}a}^{j}+c_{k}N_{\text{b}a}}{d_{\text{m}}}\leq \frac{\sum_{j}\left(\left(b_{jk}\left(\sum_{i}\left(\left(a_{ij}A_{\text{f}}^{i}\right)/\left(d_{\text{f}}+\sum_{j}a_{ij}\right)\right)+\left(\left(l_{j}A_{\text{b}}\right)/\left(d_{\text{b}}+\sum_{j}l_{j}+\sum_{k}c_{k}\right)\right)\right)\right)/\left(d_{\text{p}}+\sum_{k}b_{jk}\right)\right)+\left(\left(c_{k}A_{\text{b}}\right)/\left(d_{\text{b}}+\sum_{j}l_{j}+\sum_{k}c_{k}\right)\right)}{d_{\text{m}}}=W_{\text{m}a}^{k}. \end{array}$$

The variation of the number of human *N*_h_(*t*) is

(10) $$\begin{array}{c} \frac{dN_{\text{h}}\left(t\right)}{dt}=A_{\text{h}}-d_{\text{h}}N_{\text{h}}-\alpha_{\text{h}}I_{\text{h}}, \end{array}$$

and thus,

(11) $$\begin{array}{c} N_{\text{h}}\left(t\right)\leq \frac{A_{\text{h}}}{d_{\text{h}}}. \end{array}$$

For convenience, we denote the positive solution (*S*_f*a*_^1^,…, *S*_f*a*_^*L*^, *I*_f*a*_^1^,…, *I*_f*a*_^*L*^, *S*_b*a*_, *I*_b*a*_, *S*_p*a*_^1^,…, *S*_p*a*_^*M*^, *I*_p*a*_^1^,…, *I*_p*a*_^*M*^, *S*_m*a*_^1^,…, *S*_m*a*_^*k*^, *I*_m*a*_^1^,…, *I*_m*a*_^*k*^, *S*_h_, *I*_h_) of system (1) by (*S*, *I*).

Let *G*≔{(*S*, *I*) ∈ *R*_+_^2(*L*+*M*+*K*)+4^, *S*_f*a*_^*i*^+*I*_f*a*_^*i*^ ≤ *W*_f*a*_^*i*^, *S*_b*a*_+*I*_b*a*_ ≤ *W*_b*a*_, *S*_p*a*_^*j*^+*I*_p*a*_^*j*^ ≤ *W*_p*a*_^*j*^, *S*_m*a*_^*k*^+*I*_m*a*_^*k*^ ≤ *W*_m*a*_^*k*^, *S*_h_+*I*_h_ ≤ (*A*_h_/*d*_h_)}, then *G* is a positively invariant for system (1).

In order to find the disease-free equilibrium of system (1), we consider

(12) $$\begin{array}{c} \left\{\begin{array}{c} \frac{dS_{\text{f}a}^{i}\left(t\right)}{dt}=A_{\text{f}}^{i}-d_{\text{f}}S_{\text{f}a}^{i}-\underset{j}{\sum }a_{ij}S_{\text{f}a}^{i},\\ \frac{dS_{\text{b}a}\left(t\right)}{dt}=A_{\text{b}}-d_{\text{b}}S_{\text{b}a}-\underset{j}{\sum }l_{j}S_{\text{b}a}-\underset{k}{\sum }c_{k}S_{\text{b}a},\\ \frac{dS_{\text{p}a}^{j}\left(t\right)}{dt}=\underset{i}{\sum }a_{ij}S_{\text{f}a}^{i}+l_{j}S_{\text{b}a}-d_{\text{p}}S_{\text{p}a}^{j}-\underset{k}{\sum }b_{jk}S_{\text{p}a}^{j},\\ \frac{dS_{\text{m}a}^{k}\left(t\right)}{dt}=\underset{j}{\sum }b_{jk}S_{\text{p}a}^{j}+c_{k}S_{\text{b}a}-d_{\text{m}}S_{\text{m}a}^{k},\\ \frac{dS_{\text{h}}\left(t\right)}{dt}=A_{\text{h}}-d_{\text{h}}S_{\text{h}}. \end{array}\right. \end{array}$$

System (12) has the unique positive equilibrium $S^{0}=\left(\underset{L}{\underset{︸}{S_{\text{f}a}^{i0}}},S_{\text{b}a}^{0},\underset{M}{\underset{︸}{S_{\text{p}a}^{j0}}},\underset{K}{\underset{︸}{S_{\text{m}a}^{k0}}},S_{\text{h}}^{0}\right)$, where *S*_f*a*_^*i*0^=*A*_f_^*i*^/(*d*_f_+∑_*j*_*a*_*ij*_), *S*_b*a*_^0^=*A*_b_/(*d*_b_+∑_*j*_*l*_*j*_+∑_*k*_*c*_*k*_), *S*_p*a*_^*j*0^=(∑_*i*_*a*_*ij*_*S*_f*a*_^*i*0^+*l*_*j*_*S*_b*a*_^0^)/(*d*_p_+∑_*k*_*b*_*jk*_), *S*_m*a*_^*k*0^=(∑_*j*_*b*_*jk*_*S*_p*a*_^*j*0^+*c*_*k*_*S*_b*a*_^0^)/*d*_m_, and *S*_h_^0^=*A*_h_/*d*_h_. Thus, $E_{0}=\left(\underset{L}{\underset{︸}{S_{\text{f}a}^{i0}}},\underset{L}{\underset{︸}{0}},S_{\text{b}a}^{0},0,\underset{M}{\underset{︸}{S_{\text{p}a}^{j0}}},\underset{M}{\underset{︸}{0}},\underset{K}{\underset{︸}{S_{\text{m}a}^{k0}}},\underset{K}{\underset{︸}{0}},S_{\text{h}}^{0},0\right)$ is the disease-free equilibrium of system (1).

According to the concepts of the next generation matrix and reproduction number presented in [23, 24], we define

(13) $$\begin{array}{c} F=\left(\begin{array}{cccc} F_{11} & 0 & 0 & 0\\ 0 & F_{22} & 0 & 0\\ 0 & 0 & F_{33} & 0\\ 0 & 0 & 0 & F_{44} \end{array}\right),\\ V=\left(\begin{array}{cccc} V_{11} & 0 & 0 & 0\\ 0 & V_{22} & 0 & 0\\ 0 & 0 & V_{33} & 0\\ 0 & 0 & 0 & V_{44} \end{array}\right), \end{array}$$

where

(14) $$\begin{array}{c} F_{11}=\left(\begin{array}{cccc} \beta_{\text{f}}S_{\text{f}a}^{10} & 0 & \cdots & 0\\ 0 & \beta_{\text{f}}S_{\text{f}a}^{20} & \cdots & 0\\ \vdots & \vdots & \cdots & \vdots \\ 0 & 0 & \cdots & \beta_{\text{f}}S_{\text{f}a}^{L0} \end{array}\right),\\ F_{22}=\left(\beta_{\text{b}}S_{\text{b}a}^{0}\right),\\ F_{33}=\left(\begin{array}{cccc} \beta_{p}S_{pa}^{10} & 0 & \cdots & 0\\ 0 & \beta_{p}S_{pa}^{20} & \cdots & 0\\ \vdots & \vdots & \cdots & \vdots \\ 0 & 0 & \cdots & \beta_{p}S_{pa}^{M0} \end{array}\right),\\ F_{44}=\left(\begin{array}{cccc} \beta_{\text{m}}S_{\text{m}a}^{10} & 0 & \cdots & 0\\ 0 & \beta_{\text{m}}S_{\text{m}a}^{20} & \cdots & 0\\ \vdots & \vdots & \cdots & \vdots \\ 0 & 0 & \cdots & \beta_{\text{f}}S_{\text{f}a}^{L0} \end{array}\right),\\ V_{11}=\left(\begin{array}{cccc} d_{\text{f}}+\alpha_{\text{f}}+\underset{j}{\sum }a_{ij} & 0 & \cdots & 0\\ 0 & d_{\text{f}}+\alpha_{\text{f}}+\underset{j}{\sum }a_{ij} & \cdots & 0\\ \vdots & \vdots & \cdots & \vdots \\ 0 & 0 & \cdots & d_{\text{f}}+\alpha_{\text{f}}+\underset{j}{\sum }a_{ij} \end{array}\right),\\ V_{33}=\left(\begin{array}{cccc} d_{\text{p}}+\alpha_{\text{p}}+\underset{k}{\sum }b_{jk} & 0 & \cdots & 0\\ 0 & d_{\text{p}}+\alpha_{\text{p}}+\underset{k}{\sum }b_{jk} & \cdots & 0\\ \vdots & \vdots & \cdots & \vdots \\ 0 & 0 & \cdots & d_{\text{p}}+\alpha_{\text{p}}+\underset{k}{\sum }b_{jk} \end{array}\right),\\ V_{44}=\left(\begin{array}{cccc} d_{\text{m}}+\alpha_{\text{m}} & 0 & \cdots & 0\\ 0 & d_{\text{m}}+\alpha_{\text{m}} & \cdots & 0\\ \vdots & \vdots & \cdots & \vdots \\ 0 & 0 & \cdots & d_{\text{m}}+\alpha_{\text{m}} \end{array}\right),\\ V_{22}=\left(d_{\text{b}}+\alpha_{\text{b}}+\underset{j}{\sum }l_{j}+\underset{k}{\sum }c_{k}\right). \end{array}$$

Set *R*_0_=*ρ*(*FV*^−1^), where *ρ* represents the spectral radius of the matrix. Then, *R*_0_ is called the reproduction number for system (1), where

(15) $$\begin{array}{c} R_{0}=\underset{1\leq i\leq L,1\leq j\leq M,1\leq k\leq K}{\max}\left\{R_{\text{f}0}^{i},R_{\text{p}0}^{j},R_{\text{m}0}^{k},R_{\text{b}0}\right\},\\ R_{\text{f}0}^{i}=\frac{\beta_{\text{f}}S_{\text{f}a}^{i0}}{d_{\text{f}}+\alpha_{\text{f}}+\sum_{j}a_{ij}},\\ R_{\text{p}0}^{j}=\frac{\beta_{\text{p}}S_{\text{p}a}^{j0}}{d_{\text{p}}+\alpha_{\text{p}}+\sum_{k}b_{jk}},\\ R_{\text{m}0}^{k}=\frac{\beta_{\text{m}}S_{\text{m}a}^{k0}}{d_{\text{m}}+\alpha_{\text{m}}},\\ R_{\text{b}0}=\frac{\beta_{\text{b}}S_{\text{b}a}^{0}}{d_{\text{b}}+\alpha_{\text{b}}+\sum_{j}l_{j}+\sum_{k}c_{k}}. \end{array}$$

If *R*_0_ < 1, then system (1) has the disease-free equilibrium *E*_0_, and *E*_0_ is locally asymptotically stable.

***Remark 1*.**

If we do not consider backyard poultry farm, then system (1) becomes

(16) $$\begin{array}{c} \left\{\begin{array}{c} \frac{dS_{\text{f}a}^{i}\left(t\right)}{dt}=A_{\text{f}}^{i}-\beta_{\text{f}}S_{\text{f}a}^{i}I_{\text{f}a}^{i}-d_{\text{f}}S_{\text{f}a}^{i}-\underset{j}{\sum }a_{ij}S_{\text{f}a}^{i},\\ \frac{dI_{\text{f}a}^{i}\left(t\right)}{dt}=\beta_{\text{f}}S_{\text{f}a}^{i}I_{\text{f}a}^{i}-d_{\text{f}}I_{\text{f}a}^{i}-\alpha_{\text{f}}I_{\text{f}a}^{i}-\underset{j}{\sum }a_{ij}I_{\text{f}a}^{i},\;i=1,\ldots ,L,\\ \frac{dS_{\text{p}a}^{j}\left(t\right)}{dt}=\underset{i}{\sum }a_{ij}S_{\text{f}a}^{i}-\beta_{\text{p}}S_{\text{p}a}^{j}I_{\text{p}a}^{j}-d_{\text{p}}S_{\text{p}a}^{j}-\underset{k}{\sum }b_{jk}S_{\text{p}a}^{j},\\ \frac{dI_{\text{p}a}^{j}\left(t\right)}{dt}=\underset{i}{\sum }a_{ij}I_{\text{f}a}^{i}+\beta_{\text{p}}S_{\text{p}a}^{j}I_{\text{p}a}^{j}-d_{\text{p}}I_{\text{p}a}^{j}-\alpha_{\text{p}}I_{\text{p}a}^{j}-\underset{k}{\sum }b_{jk}I_{\text{p}a}^{j},\;j=1,\ldots ,M,\\ \frac{dS_{\text{m}a}^{k}\left(t\right)}{dt}=\underset{j}{\sum }b_{jk}S_{\text{p}a}^{j}-\beta_{\text{m}}S_{\text{m}a}^{k}I_{\text{m}a}^{k}-d_{\text{m}}S_{\text{m}a}^{k},\\ \frac{dI_{\text{m}a}^{k}\left(t\right)}{dt}=\underset{j}{\sum }b_{jk}I_{\text{p}a}^{j}+\beta_{\text{m}}S_{\text{m}a}^{k}I_{\text{m}a}^{k}-d_{\text{m}}I_{\text{m}a}^{k}-\alpha_{\text{m}}I_{\text{m}a}^{k},\;k=1,\ldots ,K,\\ \frac{dS_{\text{h}}\left(t\right)}{dt}=A_{\text{h}}-\underset{k}{\sum }\beta_{k\text{h}}S_{\text{h}}I_{\text{m}a}^{k}-d_{\text{h}}S_{\text{h}},\\ \frac{dI_{\text{h}}\left(t\right)}{dt}=\underset{k}{\sum }\beta_{k\text{h}}S_{\text{h}}I_{\text{m}a}^{k}-d_{\text{h}}I_{\text{h}}-\alpha_{\text{h}}I_{\text{h}}-\gamma_{\text{h}}I_{\text{h}},\\ \frac{dR_{\text{h}}\left(t\right)}{dt}=\gamma_{\text{h}}I_{\text{h}}-d_{\text{h}}R_{\text{h}}. \end{array}\right. \end{array}$$

A similar analysis is available for the above system.

## 3. Analysis of Subsystems of System (1)

Consider the poultry of the poultry farm subsystem, given by the first two equations of system (1), as follows

(17) $$\begin{array}{c} \left\{\begin{array}{c} \frac{dS_{\text{f}a}^{i}\left(t\right)}{dt}=A_{\text{f}}^{i}-\beta_{\text{f}}S_{\text{f}a}^{i}I_{\text{f}a}^{i}-d_{\text{f}}S_{\text{f}a}^{i}-\underset{j}{\sum }a_{ij}S_{\text{f}a}^{i},\\ \frac{dI_{\text{f}a}^{i}\left(t\right)}{dt}=\beta_{\text{f}}S_{\text{f}a}^{i}I_{\text{f}a}^{i}-d_{\text{f}}I_{\text{f}a}^{i}-\alpha_{\text{f}}I_{\text{f}a}^{i}-\underset{j}{\sum }a_{ij}I_{\text{f}a}^{i}. \end{array}\right. \end{array}$$

Let the right-hand side of system (17) equals to zero; when *I*_f*a*_^*i*^ ≠ 0, we obtain

(18) $$\begin{array}{c} S_{\text{f}a}^{i*}=\frac{d_{\text{f}}+\alpha_{\text{f}}+\sum_{j}a_{ij}}{\beta_{\text{f}}},\\ I_{\text{f}a}^{i*}=\frac{A_{\text{f}}^{i}\beta_{\text{f}}-\left(d_{\text{f}}+\sum_{j}a_{ij}\right)\left(d_{\text{f}}+\alpha_{\text{f}}+\sum_{j}a_{ij}\right)}{\beta_{\text{f}}\left(d_{\text{f}}+\alpha_{\text{f}}+\sum_{j}a_{ij}\right)}. \end{array}$$

If *R*_f0_^*i*^ > 1, system (17) has the positive equilibrium (*S*_f*a*_^*i∗*^, *I*_f*a*_^*i∗*^). If *R*_f0_^*i*^ < 1, system (17) has only the disease-free equilibrium (*S*_f*a*_^*i*0^, 0).

***Remark 2*.**

1. If $\underset{1\leq i\leq L}{\min}\left\{R_{\text{f0}}^{i}\right\}>1$, then each farm has the positive equilibrium.
2. If $\underset{1\leq i\leq L}{\max}\left\{R_{\text{f0}}^{i}\right\}>1$, then some of the poultry farms have the positive equilibrium, and the others have only the disease-free equilibrium.

Consider the poultry of the backyard poultry farm subsystem, given by the third and fourth equations of system (1), as follows

(19) $$\begin{array}{c} \left\{\begin{array}{c} \frac{dS_{\text{b}a}\left(t\right)}{dt}=A_{\text{b}}-\beta_{\text{b}}S_{\text{b}a}I_{\text{b}a}-d_{\text{b}}S_{\text{b}a}-\underset{j}{\sum }l_{j}S_{\text{b}a}-\underset{k}{\sum }c_{k}S_{\text{b}a},\\ \frac{dI_{\text{b}a}\left(t\right)}{dt}=\beta_{\text{b}}S_{\text{b}a}I_{\text{b}a}-d_{\text{b}}I_{\text{b}a}-\alpha_{\text{b}}I_{\text{b}a}-\underset{j}{\sum }l_{j}I_{\text{b}a}-\underset{k}{\sum }c_{k}I_{\text{b}a}. \end{array}\right. \end{array}$$

Let the right-hand side of system (19) equals to zero; when *I*_b*a*_ ≠ 0, we obtain

(20) $$\begin{array}{c} S_{\text{b}a}^{*}=\frac{d_{\text{b}}+\alpha_{\text{b}}+\sum_{j}l_{j}+\sum_{k}c_{k}}{\beta_{\text{b}}},\\ I_{\text{b}a}^{*}=\frac{A_{\text{b}}-\left(d_{\text{b}}+\sum_{j}l_{j}+\sum_{k}c_{k}\right)S_{\text{b}a}^{*}}{\beta_{\text{b}}S_{\text{b}a}^{*}}. \end{array}$$

If *R*_b0_ > 1, system (19) has the positive equilibrium (*S*_b*a*_^*i∗*^, *I*_b*a*_^*i∗*^). If *R*_b0_ < 1, system (19) has only the disease-free equilibrium (*S*_b*a*_^0^, 0).

Consider the poultry of the live-poultry wholesale market subsystem, given by the fifth and sixth equations of system (1), as follows

(21) $$\begin{array}{c} \left\{\begin{array}{c} \frac{dS_{\text{p}a}^{j}\left(t\right)}{dt}=\underset{i}{\sum }a_{ij}S_{\text{f}a}^{i}+l_{j}S_{\text{b}a}-\beta_{\text{p}}S_{\text{p}a}^{j}I_{\text{p}a}^{j}-d_{\text{p}}S_{\text{p}a}^{j}-\underset{k}{\sum }b_{jk}S_{\text{p}a}^{j},\\ \frac{dI_{\text{p}a}^{j}\left(t\right)}{dt}=\underset{i}{\sum }a_{ij}I_{\text{f}a}^{i}+l_{j}I_{\text{b}a}+\beta_{\text{p}}S_{\text{p}a}^{j}I_{\text{p}a}^{j}-d_{\text{p}}I_{\text{p}a}^{j}-\alpha_{\text{p}}I_{\text{p}a}^{j}-\underset{k}{\sum }b_{jk}I_{\text{p}a}^{j}. \end{array}\right. \end{array}$$

Let the right-hand side of system (21) equals to zero; when *I*_p*a*_^*j*^ ≠ 0, we can divide it into two cases.

If *I*_f*a*_^*i*^=0 and *I*_b*a*_=0, then we have

(22) $$\begin{array}{c} S_{\text{p}a}^{j*}=\frac{d_{\text{p}}+\alpha_{\text{p}}+\sum_{k}b_{jk}}{\beta_{\text{p}}},\\ I_{\text{p}a}^{j*}=\frac{\sum_{i}a_{ij}S_{\text{f}a}^{i0}+l_{j}S_{\text{b}a}^{0}-\left(d_{\text{p}}+\sum_{k}b_{jk}\right)S_{\text{p}a}^{j*}}{d_{\text{p}}+\alpha_{\text{p}}+\sum_{k}b_{jk}}. \end{array}$$

If *R*_p0_^*j*^ > 1, then system (21) has the positive equilibrium (*S*_p*a*_^*j∗*^, *I*_p*a*_^*j∗*^).

If *I*_f*a*_^*i*^ ≠ 0 or *I*_b*a*_ ≠ 0, then we obtain

(23) $$\begin{array}{c} I_{\text{p}a}^{j*}=\frac{\sum_{i}a_{ij}I_{\text{f}a}^{i*}+l_{j}I_{\text{b}a}^{*}}{d_{\text{p}}+\alpha_{\text{p}}+\sum_{k}b_{jk}-\beta_{\text{p}}S_{\text{p}a}^{j*}},\\ b_{1}S_{\text{p}a}^{j*2}+b_{2}S_{\text{p}a}^{j*}+b_{3}=0, \end{array}$$

where

(24) $$\begin{array}{c} b_{1}=-\beta_{\text{p}}\left(d_{\text{p}}+\underset{k}{\sum }b_{jk}\right)<0,\\ b_{2}=\beta_{\text{p}}\left(\underset{i}{\sum }a_{ij}S_{\text{f}a}^{i*}+l_{j}S_{\text{b}a}^{*}\right)+\beta_{\text{p}}\left(\underset{i}{\sum }a_{ij}I_{\text{f}a}^{i*}+l_{j}I_{\text{b}a}^{*}\right)+\left(d_{\text{p}}+\underset{k}{\sum }b_{jk}\right)\left(d_{\text{p}}+\alpha_{\text{p}}+\underset{k}{\sum }b_{jk}\right)>0,\\ b_{3}=-\left(d_{\text{p}}+\alpha_{\text{p}}+\underset{k}{\sum }b_{jk}\right)\left(\underset{i}{\sum }a_{ij}S_{\text{f}a}^{i*}+l_{j}S_{\text{b}a}^{*}\right)<0. \end{array}$$

Because *b*_2_^2^ − 4*b*_1_*b*_3_ > 0, the solutions of the above equation are

(25) $$\begin{array}{c} S_{\text{p}a1}^{j*}=\frac{-b_{2}+\sqrt{b_{2}^{2}-4b_{1}b_{3}}}{2b_{1}}>0,\\ S_{\text{p}a2}^{j*}=\frac{-b_{2}-\sqrt{b_{2}^{2}-4b_{1}b_{3}}}{2b_{1}}>0. \end{array}$$

If *R*_p0_^*j*2^=((*d*_p_+*α*_p_+∑_*k*_*b*_*jk*_)/(*β*_p_*S*_p*a*2_^*j∗*^)) > 1, system (21) has two positive equilibria (*S*_p*a*1_^*j∗*^, *I*_p*a*1_^*j∗*^) and (*S*_p*a*2_^*j∗*^, *I*_p*a*2_^*j∗*^). If *R*_p0_^*j*2^ < 1 and *R*_p0_^*j*1^=((*d*_p_+*α*_p_+∑_*k*_*b*_*jk*_)/(*β*_p_*S*_p*a*1_^*j∗*^)) > 1, system (21) has one positive equilibrium (*S*_p*a*1_^*j∗*^, *I*_p*a*1_^*j∗*^). If *R*_p0_^*j*1^ < 1, system (21) has no positive equilibrium.

Consider the poultry of the wet market (the retail live-poultry market) subsystem, given by the seventh and eighth equations of system (1), as follows:

(26) $$\begin{array}{c} \left\{\begin{array}{c} \frac{dS_{\text{m}a}^{k}\left(t\right)}{dt}=\underset{j}{\sum }b_{jk}S_{\text{p}a}^{j}+c_{k}S_{\text{b}a}-\beta_{\text{m}}S_{\text{m}a}^{k}I_{\text{m}a}^{k}-d_{\text{m}}S_{\text{m}a}^{k},\\ \frac{dI_{\text{m}a}^{k}\left(t\right)}{dt}=\underset{j}{\sum }b_{jk}I_{\text{p}a}^{j}+c_{k}I_{\text{b}a}+\beta_{\text{m}}S_{\text{m}a}^{k}I_{\text{m}a}^{k}-d_{\text{m}}I_{\text{m}a}^{k}-\alpha_{\text{m}}I_{\text{m}a}^{k}. \end{array}\right. \end{array}$$

Let the right-hand side of system (26) equals to zero, when *I*_m*a*_^*k*^ ≠ 0, we can divide it into two cases.

If *I*_p*a*_^*j*^=0 and *I*_b*a*_=0, then we have

(27) $$\begin{array}{c} S_{\text{m}a}^{k*}=\frac{d_{\text{m}}+\alpha_{\text{m}}}{\beta_{\text{m}}},\\ I_{\text{m}a}^{k*}=\frac{\sum_{j}b_{jk}S_{\text{p}a}^{j0}+c_{k}S_{\text{b}a}^{0}-d_{\text{m}}S_{\text{m}a}^{k*}}{d_{\text{m}}+\alpha_{\text{m}}}. \end{array}$$

If *R*_m0_^*k*^ > 1, then system (26) has the positive equilibrium (*S*_m*a*_^*k∗*^, *I*_m*a*_^*k∗*^).

If *I*_p*a*_^*j*^ ≠ 0 or *I*_b*a*_ ≠ 0, then we have

(28) $$\begin{array}{c} I_{\text{m}a}^{k*}=\frac{\sum_{j}b_{jk}I_{\text{p}a}^{j*}+c_{k}I_{\text{b}a}^{*}}{d_{\text{m}}+\alpha_{\text{m}}-\beta_{\text{m}}S_{\text{m}a}^{k*}},\\ g_{1}S_{\text{m}a}^{k*2}+g_{2}S_{\text{m}a}^{k*}+g_{3}=0. \end{array}$$

where

(29) $$\begin{array}{c} g_{1}=-d_{\text{m}}\beta_{\text{m}}<0,\\ g_{2}=\beta_{\text{m}}\left(\underset{j}{\sum }b_{jk}S_{\text{p}a}^{j*}+c_{k}S_{\text{b}a}^{*}\right)+\beta_{\text{m}}\left(\underset{j}{\sum }b_{jk}I_{\text{p}a}^{j*}+c_{k}I_{\text{b}a}^{*}\right)+d_{\text{m}}\left(d_{\text{m}}+\alpha_{\text{m}}\right)>0,\\ g_{3}=-\left(d_{\text{m}}+\alpha_{\text{m}}\right)\left(\underset{j}{\sum }b_{jk}S_{\text{p}a}^{j*}+c_{k}S_{\text{b}a}^{*}\right)<0. \end{array}$$

Because *g*_2_^2^ − 4*g*_1_*g*_3_ > 0, the solutions of the above equation are

(30) $$\begin{array}{c} S_{\text{m}a1}^{k*}=\frac{-g_{2}+\sqrt{g_{2}^{2}-4g_{1}g_{3}}}{2g_{1}}>0,\\ S_{\text{m}a2}^{k*}=\frac{-g_{2}-\sqrt{g_{2}^{2}-4g_{1}g_{3}}}{2g_{1}}>0. \end{array}$$

If *R*_m0_^*k*2^=((*d*_m_+*α*_m_)/(*β*_m_*S*_m*a*2_^*k∗*^)) > 1, system (26) has two positive equilibria (*S*_m*a*1_^*k∗*^, *I*_m*a*1_^*k∗*^) and (*S*_m*a*2_^*k∗*^, *I*_m*a*2_^*k∗*^). If *R*_m0_^*k*2^ < 1 and *R*_m0_^*k*1^=((*d*_m_+*α*_m_)/(*β*_m_*S*_m*a*1_^*k∗*^)) > 1, system (26) has one positive equilibrium (*S*_m*a*1_^*k∗*^, *I*_m*a*1_^*k∗*^). If *R*_m0_^*k*1^ < 1, system (26) has no positive equilibrium.

Consider the human subsystem, given by the last three equations of system (1), as follows:

(31) $$\begin{array}{c} \left\{\begin{array}{c} \frac{dS_{\text{h}}\left(t\right)}{dt}=A_{\text{h}}-\underset{k}{\sum }\beta_{k\text{h}}S_{\text{h}}I_{\text{m}a}^{k}-\beta_{\text{h}}S_{\text{h}}I_{\text{b}a}-d_{\text{h}}S_{\text{h}},\\ \frac{dI_{\text{h}}\left(t\right)}{dt}=\underset{k}{\sum }\beta_{k\text{h}}S_{\text{h}}I_{\text{m}a}^{k}+\beta_{\text{h}}S_{\text{h}}I_{\text{b}a}-d_{\text{h}}I_{\text{h}}-\alpha_{\text{h}}I_{\text{h}}-\gamma_{\text{h}}I_{\text{h}},\\ \frac{dR_{\text{h}}\left(t\right)}{dt}=\gamma_{\text{h}}I_{\text{h}}-d_{\text{h}}R_{\text{h}}. \end{array}\right. \end{array}$$

Since the first two equations of system (31) are independent of the variable *R*_h_, we only need to analyze the first two equations of system (31). Let the right-hand side of system (31) equals to zero, when *I*_h_ ≠ 0, if *I*_m*a*_^*k*^ ≠ 0 or *I*_b*a*_ ≠ 0, then we have

(32) $$\begin{array}{c} S_{\text{h}}^{*}=\frac{A_{\text{h}}}{\sum_{k}\beta_{k\text{h}}I_{\text{m}a}^{k*}+\beta_{\text{h}}I_{\text{b}a}^{*}+d_{\text{h}}},\\ I_{\text{h}}^{*}=\frac{\left(\sum_{k}\beta_{k\text{h}}I_{\text{m}a}^{k*}+\beta_{\text{h}}I_{\text{b}a}^{*}\right)S_{\text{h}}}{d_{\text{h}}+\alpha_{\text{h}}+\gamma_{\text{h}}}. \end{array}$$

## 4. Analysis of the Full System (1)

We analyze the following equivalent system:

(33) $$\begin{array}{c} \left\{\begin{array}{c} \frac{dS_{\text{f}a}^{i}\left(t\right)}{dt}=A_{\text{f}}^{i}-\beta_{\text{f}}S_{\text{f}a}^{i}I_{\text{f}a}^{i}-d_{\text{f}}S_{\text{f}a}^{i}-\underset{j}{\sum }a_{ij}S_{\text{f}a}^{i},\\ \frac{dI_{\text{f}a}^{i}\left(t\right)}{dt}=\beta_{\text{f}}S_{\text{f}a}^{i}I_{\text{f}a}^{i}-d_{\text{f}}I_{\text{f}a}^{i}-\alpha_{\text{f}}I_{\text{f}a}^{i}-\underset{j}{\sum }a_{ij}I_{\text{f}a}^{i},\\ \frac{dS_{\text{b}a}\left(t\right)}{dt}=A_{\text{b}}-\beta_{\text{b}}S_{\text{b}a}I_{\text{b}a}-d_{\text{b}}S_{\text{b}a}-\underset{j}{\sum }l_{j}S_{\text{b}a}-\underset{k}{\sum }c_{k}S_{\text{b}a},\\ \frac{dI_{\text{b}a}\left(t\right)}{dt}=\beta_{\text{b}}S_{\text{b}a}I_{\text{b}a}-d_{\text{b}}I_{\text{b}a}-\alpha_{\text{b}}I_{\text{b}a}-\underset{j}{\sum }l_{j}I_{\text{b}a}-\underset{k}{\sum }c_{k}I_{\text{b}a},\\ \frac{dS_{\text{p}a}^{j}\left(t\right)}{dt}=\underset{i}{\sum }a_{ij}S_{\text{f}a}^{i}+l_{j}S_{\text{b}a}-\beta_{\text{p}}S_{\text{p}a}^{j}I_{\text{p}a}^{j}-d_{\text{p}}S_{\text{p}a}^{j}-\underset{k}{\sum }b_{jk}S_{\text{p}a}^{j},\\ \frac{dI_{\text{p}a}^{j}\left(t\right)}{dt}=\underset{i}{\sum }a_{ij}I_{\text{f}a}^{i}+l_{j}I_{\text{b}a}+\beta_{\text{p}}S_{\text{p}a}^{j}I_{\text{p}a}^{j}-d_{\text{p}}I_{\text{p}a}^{j}-\alpha_{\text{p}}I_{\text{p}a}^{j}-\underset{k}{\sum }b_{jk}I_{\text{p}a}^{j},\\ \frac{dS_{\text{m}a}^{k}\left(t\right)}{dt}=\underset{j}{\sum }b_{jk}S_{\text{p}a}^{j}+c_{k}S_{\text{b}a}-\beta_{\text{m}}S_{\text{m}a}^{k}I_{\text{m}a}^{k}-d_{\text{m}}S_{\text{m}a}^{k},\\ \frac{dI_{\text{m}a}^{k}\left(t\right)}{dt}=\underset{j}{\sum }b_{jk}I_{\text{p}a}^{j}+c_{k}I_{\text{b}a}+\beta_{\text{m}}S_{\text{m}a}^{k}I_{\text{m}a}^{k}-d_{\text{m}}I_{\text{m}a}^{k}-\alpha_{\text{m}}I_{\text{m}a}^{k},\\ \frac{dS_{\text{h}}\left(t\right)}{dt}=A_{\text{h}}-\underset{k}{\sum }\beta_{k\text{h}}S_{\text{h}}I_{\text{m}a}^{k}-\beta_{\text{h}}S_{\text{h}}I_{\text{b}a}-d_{\text{h}}S_{\text{h}},\\ \frac{dI_{\text{h}}\left(t\right)}{dt}=\underset{k}{\sum }\beta_{k\text{h}}S_{\text{h}}I_{\text{m}a}^{k}+\beta_{\text{h}}S_{\text{h}}I_{\text{b}a}-d_{\text{h}}I_{\text{h}}-\alpha_{\text{h}}I_{\text{h}}-\gamma_{\text{h}}I_{\text{h}}. \end{array}\right. \end{array}$$

For the sake of discussion, without loss of generality, we assume that a node has at least one link with the nodes in the next layer. So we have the following cases.

***Case 1*.**

If $R_{0}=\underset{1\leq i\leq L,1\leq j\leq M,1\leq k\leq K}{\max}\left\{R_{\text{f}0}^{i},R_{\text{p}0}^{j},R_{\text{m}0}^{k},R_{\text{b}0}\right\}<1$, system (33) has only the disease-free equilibrium $E_{0}=\left(\underset{L}{\underset{︸}{S_{\text{f}a}^{i0}}},\underset{L}{\underset{︸}{0}},S_{\text{b}a}^{0},0,\underset{M}{\underset{︸}{S_{\text{p}a}^{j0}}},\underset{M}{\underset{︸}{0}},\underset{K}{\underset{︸}{S_{\text{m}a}^{k0}}},\underset{K}{\underset{︸}{0}},S_{\text{h}}^{0},0\right)$. Namely, when all poultry has no avian influenza, human will not be infected with avian influenza.

***Case 2*.**

If $\underset{1\leq i\leq L,1\leq j\leq M}{\max}\left\{R_{\text{f}0}^{i},R_{\text{p}0}^{j}\right\}<1$, *R*_b0_ < 1, and $\underset{1\leq k\leq K}{\min}\left\{R_{\text{m}0}^{k}\right\}>1$, system (33) has the boundary equilibrium

(34) $$\begin{array}{c} E^{*}=\left(\underset{L}{\underset{︸}{S_{\text{f}a}^{i0}}},\underset{L}{\underset{︸}{0}},S_{\text{b}a}^{0},0,\underset{M}{\underset{︸}{S_{\text{p}a}^{j0}}},\underset{M}{\underset{︸}{0}},\underset{K}{\underset{︸}{S_{\text{m}a}^{k*}}},\underset{K}{\underset{︸}{I_{\text{m}a}^{k*}}},S_{\text{h}}^{\text{m}*},I_{\text{h}}^{\text{m}*}\right). \end{array}$$

This shows that avian influenza A (H7N9) virus is most likely transmitted from the retail live-poultry market to humans when poultry has no disease in other types of farms.

***Case 3*.**

If $\underset{1\leq i\leq L}{\max}\left\{R_{\text{f}0}^{i}\right\}<1$, *R*_b0_ < 1, and $\underset{1\leq j\leq M}{\min}\left\{R_{\text{p}0}^{j}\right\}>1$, system (33) has the boundary equilibrium as described next.

If $\underset{1\leq k\leq K}{\min}\left\{R_{\text{m}0}^{k1}\right\}>1$ and $\underset{1\leq k\leq K}{\max}\left\{R_{\text{m}0}^{k2}\right\}<1$, system (33) has one boundary equilibrium:

(35) $$\begin{array}{c} E^{*}=\left(\underset{L}{\underset{︸}{S_{\text{f}a}^{i0}}},\underset{L}{\underset{︸}{0}},S_{\text{b}a}^{0},0,\underset{M}{\underset{︸}{S_{\text{p}a}^{j*}}},\underset{M}{\underset{︸}{I_{\text{p}a}^{j*}}},\underset{K}{\underset{︸}{S_{\text{m}a1}^{k\text{p}*}}},\underset{K}{\underset{︸}{I_{\text{m}a1}^{k\text{p}*}}},S_{\text{h}1}^{\text{pm}*},I_{\text{h}1}^{\text{pm}*}\right). \end{array}$$

If $\underset{1\leq k\leq K}{\min}\left\{R_{\text{m}0}^{k2}\right\}>1$, system (33) has two boundary equilibria:

(36) $$\begin{array}{c} E^{1*}=\left(\underset{L}{\underset{︸}{S_{\text{f}a}^{i0}}},\underset{L}{\underset{︸}{0}},S_{\text{b}a}^{0},0,\underset{M}{\underset{︸}{S_{\text{p}a}^{j*}}},\underset{M}{\underset{︸}{I_{\text{p}a}^{j*}}},\underset{K}{\underset{︸}{S_{\text{m}a1}^{k\text{p}*}}},\underset{K}{\underset{︸}{I_{\text{m}a1}^{k\text{p}*}}},S_{\text{h}1}^{\text{pm}*},I_{\text{h}1}^{\text{pm}*}\right),\\ E^{2*}=\left(\underset{L}{\underset{︸}{S_{\text{f}a}^{i0}}},\underset{L}{\underset{︸}{0}},S_{\text{b}a}^{0},0,\underset{M}{\underset{︸}{S_{\text{p}a}^{j*}}},\underset{M}{\underset{︸}{I_{\text{p}a}^{j*}}},\underset{K}{\underset{︸}{S_{\text{m}a2}^{k\text{p}*}}},\underset{K}{\underset{︸}{I_{\text{m}a2}^{k\text{p}*}}},S_{\text{h}2}^{\text{pm}*},I_{\text{h}2}^{\text{pm}*}\right). \end{array}$$

This shows that avian influenza A (H7N9) virus is most likely transmitted from the secondary wholesale market to the retail live-poultry market and then to humans [6, 7]. And there may be two boundary equilibria.

***Case 4*.**

If $\underset{1\leq i\leq L}{\max}\left\{R_{\text{f}0}^{i}\right\}<1$ and *R*_b0_ > 1, system (33) has the boundary equilibrium as described next.

If $\underset{1\leq j\leq M}{\min}\left\{R_{\text{p}0}^{j1}\right\}>1$, $\underset{1\leq j\leq M}{\max}\left\{R_{\text{p}0}^{j2}\right\}<1$, $\underset{1\leq k\leq K}{\min}\left\{R_{\text{m}0}^{k1}\right\}>1$, and $\underset{1\leq k\leq K}{\max}\left\{R_{\text{m}0}^{k2}\right\}<1$, system (33) has one boundary equilibrium:

(37) $$\begin{array}{c} E^{*}=\left(\underset{L}{\underset{︸}{S_{\text{f}a}^{i0}}},\underset{L}{\underset{︸}{0}},S_{\text{b}a}^{*},I_{\text{b}a}^{*},\underset{M}{\underset{︸}{S_{\text{p}a1}^{j\text{b}*}}},\underset{M}{\underset{︸}{I_{\text{p}a1}^{j\text{b}*}}},\underset{K}{\underset{︸}{S_{\text{m}a11}^{k\text{bp}*}}},\underset{K}{\underset{︸}{I_{\text{m}a11}^{k\text{bp}*}}},S_{\text{h}11}^{\text{bpm}*},I_{\text{h}11}^{\text{bpm}*}\right). \end{array}$$

If $\underset{1\leq j\leq M}{\min}\left\{R_{\text{p}0}^{j1}\right\}>1$, $\underset{1\leq j\leq M}{\max}\left\{R_{\text{p}0}^{j2}\right\}<1$, and $\underset{1\leq k\leq K}{\min}\left\{R_{\text{m}0}^{k2}\right\}>1$, system (33) has two boundary equilibria:

(38) $$\begin{array}{c} E^{1*}=\left(\underset{L}{\underset{︸}{S_{\text{f}a}^{i0}}},\underset{L}{\underset{︸}{0}},S_{\text{b}a}^{*},I_{\text{b}a}^{*},\underset{M}{\underset{︸}{S_{\text{p}a1}^{j\text{b}*}}},\underset{M}{\underset{︸}{I_{\text{p}a1}^{j\text{b}*}}},\underset{K}{\underset{︸}{S_{\text{m}a11}^{k\text{bp}*}}},\underset{K}{\underset{︸}{I_{\text{m}a11}^{k\text{bp}*}}},S_{\text{h}11}^{\text{bpm}*},I_{\text{h}11}^{\text{bpm}*}\right),\\ E^{2*}=\left(\underset{L}{\underset{︸}{S_{\text{f}a}^{i0}}},\underset{L}{\underset{︸}{0}},S_{\text{b}a}^{*},I_{\text{b}a}^{*},\underset{M}{\underset{︸}{S_{\text{p}a1}^{j\text{b}*}}},\underset{M}{\underset{︸}{I_{\text{p}a1}^{j\text{b}*}}},\underset{K}{\underset{︸}{S_{\text{m}a12}^{k\text{bp}*}}},\underset{K}{\underset{︸}{I_{\text{m}a12}^{k\text{bp}*}}},S_{\text{h}12}^{\text{bpm}*},I_{\text{h}12}^{\text{bpm}*}\right). \end{array}$$

If $\underset{1\leq j\leq M}{\min}\left\{R_{\text{p}0}^{j2}\right\}>1$, $\underset{1\leq k\leq K}{\max}\left\{R_{\text{m}0}^{k2}\right\}<1$, and $\underset{1\leq k\leq K}{\min}\left\{R_{\text{m}0}^{k1}\right\}>1$, system (33) has two boundary equilibria:

(39) $$\begin{array}{c} E^{1*}=\left(\underset{L}{\underset{︸}{S_{\text{f}a}^{i0}}},\underset{L}{\underset{︸}{0}},S_{\text{b}a}^{*},I_{\text{b}a}^{*},\underset{M}{\underset{︸}{S_{\text{p}a1}^{j\text{b}*}}},\underset{M}{\underset{︸}{I_{\text{p}a1}^{j\text{b}*}}},\underset{K}{\underset{︸}{S_{\text{m}a11}^{k\text{bp}*}}},\underset{K}{\underset{︸}{I_{\text{m}a11}^{k\text{bp}*}}},S_{\text{h}11}^{\text{bpm}*},I_{\text{h}11}^{\text{bpm}*}\right),\\ E^{2*}=\left(\underset{L}{\underset{︸}{S_{\text{f}a}^{i0}}},\underset{L}{\underset{︸}{0}},S_{\text{b}a}^{*},I_{\text{b}a}^{*},\underset{M}{\underset{︸}{S_{\text{p}a2}^{j\text{b}*}}},\underset{M}{\underset{︸}{I_{\text{p}a2}^{j\text{b}*}}},\underset{K}{\underset{︸}{S_{\text{m}a21}^{k\text{bp}*}}},\underset{K}{\underset{︸}{I_{\text{m}a21}^{k\text{bp}*}}},S_{\text{h}21}^{\text{bpm}*},I_{\text{h}21}^{\text{bpm}*}\right). \end{array}$$

If $\underset{1\leq j\leq M}{\min}\left\{R_{\text{p}0}^{j2}\right\}>1$ and $\underset{1\leq k\leq K}{\min}\left\{R_{\text{m}0}^{k2}\right\}>1$, system (33) has four boundary equilibria:

(40) $$\begin{array}{c} E^{1*}=\left(\underset{L}{\underset{︸}{S_{\text{f}a}^{i0}}},\underset{L}{\underset{︸}{0}},S_{\text{b}a}^{*},I_{\text{b}a}^{*},\underset{M}{\underset{︸}{S_{\text{p}a1}^{j\text{b}*}}},\underset{M}{\underset{︸}{I_{\text{p}a1}^{j\text{b}*}}},\underset{K}{\underset{︸}{S_{\text{m}a11}^{k\text{bp}*}}},\underset{K}{\underset{︸}{I_{\text{m}a11}^{k\text{bp}*}}},S_{\text{h}11}^{\text{bpm}*},I_{\text{h}11}^{\text{bpm}*}\right),\\ E^{2*}=\left(\underset{L}{\underset{︸}{S_{\text{f}a}^{i0}}},\underset{L}{\underset{︸}{0}},S_{\text{b}a}^{*},I_{\text{b}a}^{*},\underset{M}{\underset{︸}{S_{\text{p}a1}^{j\text{b}*}}},\underset{M}{\underset{︸}{I_{\text{p}a1}^{j\text{b}*}}},\underset{K}{\underset{︸}{S_{\text{m}a12}^{k\text{bp}*}}},\underset{K}{\underset{︸}{I_{\text{m}a12}^{k\text{bp}*}}},S_{\text{h}12}^{\text{bpm}*},I_{\text{h}12}^{\text{bpm}*}\right),\\ E^{3*}=\left(\underset{L}{\underset{︸}{S_{\text{f}a}^{i0}}},\underset{L}{\underset{︸}{0}},S_{\text{b}a}^{*},I_{\text{b}a}^{*},\underset{M}{\underset{︸}{S_{\text{p}a2}^{j\text{b}*}}},\underset{M}{\underset{︸}{I_{\text{p}a2}^{j\text{b}*}}},\underset{K}{\underset{︸}{S_{\text{m}a21}^{k\text{bp}*}}},\underset{K}{\underset{︸}{I_{\text{m}a21}^{k\text{bp}*}}},S_{\text{h}21}^{\text{bpm}*},I_{\text{h}21}^{\text{bpm}*}\right),\\ E^{4*}=\left(\underset{L}{\underset{︸}{S_{\text{f}a}^{i0}}},\underset{L}{\underset{︸}{0}},S_{\text{b}a}^{*},I_{\text{b}a}^{*},\underset{M}{\underset{︸}{S_{\text{p}a2}^{j\text{b}*}}},\underset{M}{\underset{︸}{I_{\text{p}a2}^{j\text{b}*}}},\underset{K}{\underset{︸}{S_{\text{m}a22}^{k\text{bp}*}}},\underset{K}{\underset{︸}{I_{\text{m}a22}^{k\text{bp}*}}},S_{\text{h}22}^{\text{bpm}*},I_{\text{h}22}^{\text{bpm}*}\right). \end{array}$$

When the poultry of poultry farms has no avian influenza, and the poultry of backyard poultry farm has avian influenza, we can obtain four cases. In four cases, human is most likely transmitted from the backyard poultry farm to the secondary wholesale market then to the retail live-poultry market, and finally to humans, or direct transmission from backyard poultry to humans.

***Case 5*.**

If $\underset{1\leq i\leq L}{\min}\left\{R_{\text{f}0}^{i}\right\}>1$ and *R*_b0_ < 1, system (33) has the boundary equilibrium as described next.

If $\underset{1\leq j\leq M}{\min}\left\{R_{\text{p}0}^{j1}\right\}>1$, $\underset{1\leq j\leq M}{\max}\left\{R_{\text{p}0}^{j2}\right\}<1$, $\underset{1\leq k\leq K}{\min}\left\{R_{\text{m}0}^{k1}\right\}>1$, and $\underset{1\leq k\leq K}{\max}\left\{R_{\text{m}0}^{k2}\right\}<1$, system (33) has one boundary equilibrium:

(41) $$\begin{array}{c} E^{*}=\left(\underset{L}{\underset{︸}{S_{\text{f}a}^{i*}}},\underset{L}{\underset{︸}{I_{\text{f}a}^{i*}}},S_{\text{b}a}^{0},0,\underset{M}{\underset{︸}{S_{\text{p}a1}^{j\text{f}*}}},\underset{M}{\underset{︸}{I_{\text{p}a1}^{j\text{f}*}}},\underset{K}{\underset{︸}{S_{\text{m}a11}^{k\text{fp}*}}},\underset{K}{\underset{︸}{I_{\text{m}a11}^{k\text{fp}*}}},S_{\text{h}11}^{\text{fpm}*},I_{\text{h}11}^{\text{fpm}*}\right). \end{array}$$

If $\underset{1\leq j\leq M}{\min}\left\{R_{\text{p}0}^{j1}\right\}>1$, $\underset{1\leq j\leq M}{\max}\left\{R_{\text{p}0}^{j2}\right\}<1$, and $\underset{1\leq k\leq K}{\min}\left\{R_{\text{m}0}^{k2}\right\}>1$, system (33) has two boundary equilibria:

(42) $$\begin{array}{c} E^{1*}=\left(\underset{L}{\underset{︸}{S_{\text{f}a}^{i*}}},\underset{L}{\underset{︸}{I_{\text{f}a}^{i*}}},S_{\text{b}a}^{0},0,\underset{M}{\underset{︸}{S_{\text{p}a1}^{j\text{f}*}}},\underset{M}{\underset{︸}{I_{\text{p}a1}^{j\text{f}*}}},\underset{K}{\underset{︸}{S_{\text{m}a11}^{k\text{fp}*}}},\underset{K}{\underset{︸}{I_{\text{m}a11}^{k\text{fp}*}}},S_{\text{h}11}^{\text{fpm}*},I_{\text{h}11}^{\text{fpm}*}\right),\\ E^{2*}=\left(\underset{L}{\underset{︸}{S_{\text{f}a}^{i*}}},\underset{L}{\underset{︸}{I_{\text{f}a}^{i*}}},S_{\text{b}a}^{0},0,\underset{M}{\underset{︸}{S_{\text{p}a1}^{j\text{f}*}}},\underset{M}{\underset{︸}{I_{\text{p}a1}^{j\text{f}*}}},\underset{K}{\underset{︸}{S_{\text{m}a12}^{k\text{fp}*}}},\underset{K}{\underset{︸}{I_{\text{m}a12}^{k\text{fp}*}}},S_{\text{h}12}^{\text{fpm}*},I_{\text{h}12}^{\text{fpm}*}\right). \end{array}$$

If $\underset{1\leq j\leq M}{\min}\left\{R_{\text{p}0}^{j2}\right\}>1$, $\underset{1\leq k\leq K}{\max}\left\{R_{\text{m}0}^{k2}\right\}<1$, and $\underset{1\leq k\leq K}{\min}\left\{R_{\text{m}0}^{k1}\right\}>1$, system (33) has two boundary equilibria:

(43) $$\begin{array}{c} E^{1*}=\left(\underset{L}{\underset{︸}{S_{\text{f}a}^{i*}}},\underset{L}{\underset{︸}{I_{\text{f}a}^{i*}}},S_{\text{b}a}^{0},0,\underset{M}{\underset{︸}{S_{\text{p}a1}^{j\text{f}*}}},\underset{M}{\underset{︸}{I_{\text{p}a1}^{j\text{f}*}}},\underset{K}{\underset{︸}{S_{\text{m}a11}^{k\text{fp}*}}},\underset{K}{\underset{︸}{I_{\text{m}a11}^{k\text{fp}*}}},S_{\text{h}11}^{\text{fpm}*},I_{\text{h}11}^{\text{fpm}*}\right),\\ E^{2*}=\left(\underset{L}{\underset{︸}{S_{\text{f}a}^{i*}}},\underset{L}{\underset{︸}{I_{\text{f}a}^{i*}}},S_{\text{b}a}^{0},0,\underset{M}{\underset{︸}{S_{\text{p}a2}^{j\text{f}*}}},\underset{M}{\underset{︸}{I_{\text{p}a2}^{j\text{f}*}}},\underset{K}{\underset{︸}{S_{\text{m}a21}^{k\text{fp}*}}},\underset{K}{\underset{︸}{I_{\text{m}a21}^{k\text{fp}*}}},S_{\text{h}21}^{\text{fpm}*},I_{\text{h}21}^{\text{fpm}*}\right). \end{array}$$

If $\underset{1\leq j\leq M}{\min}\left\{R_{\text{p}0}^{j2}\right\}>1$ and $\underset{1\leq k\leq K}{\min}\left\{R_{\text{m}0}^{k2}\right\}>1$, system (33) has four boundary equilibria

(44) $$\begin{array}{c} E^{1*}=\left(\underset{L}{\underset{︸}{S_{\text{f}a}^{i*}}},\underset{L}{\underset{︸}{I_{\text{f}a}^{i*}}},S_{\text{b}a}^{0},0,\underset{M}{\underset{︸}{S_{\text{p}a1}^{j\text{f}*}}},\underset{M}{\underset{︸}{I_{\text{p}a1}^{j\text{f}*}}},\underset{K}{\underset{︸}{S_{\text{m}a11}^{k\text{fp}*}}},\underset{K}{\underset{︸}{I_{\text{m}a11}^{k\text{fp}*}}},S_{\text{h}11}^{\text{fpm}*},I_{\text{h}11}^{\text{fpm}*}\right),\\ E^{2*}=\left(\underset{L}{\underset{︸}{S_{\text{f}a}^{i*}}},\underset{L}{\underset{︸}{I_{\text{f}a}^{i*}}},S_{\text{b}a}^{0},0,\underset{M}{\underset{︸}{S_{\text{p}a1}^{j\text{f}*}}},\underset{M}{\underset{︸}{I_{\text{p}a1}^{j\text{f}*}}},\underset{K}{\underset{︸}{S_{\text{m}a12}^{k\text{fp}*}}},\underset{K}{\underset{︸}{I_{\text{m}a12}^{k\text{fp}*}}},S_{\text{h}12}^{\text{fpm}*},I_{\text{h}12}^{\text{fpm}*}\right),\\ E^{3*}=\left(\underset{L}{\underset{︸}{S_{\text{f}a}^{i*}}},\underset{L}{\underset{︸}{I_{\text{f}a}^{i*}}},S_{\text{b}a}^{0},0,\underset{M}{\underset{︸}{S_{\text{p}a2}^{j\text{f}*}}},\underset{M}{\underset{︸}{I_{\text{p}a2}^{j\text{f}*}}},\underset{K}{\underset{︸}{S_{\text{m}a21}^{k\text{fp}*}}},\underset{K}{\underset{︸}{I_{\text{m}a21}^{k\text{fp}*}}},S_{\text{h}21}^{\text{fpm}*},I_{\text{h}21}^{\text{fpm}*}\right),\\ E^{4*}=\left(\underset{L}{\underset{︸}{S_{\text{f}a}^{i*}}},\underset{L}{\underset{︸}{I_{\text{f}a}^{i*}}},S_{\text{b}a}^{0},0,\underset{M}{\underset{︸}{S_{\text{p}a2}^{j\text{f}*}}},\underset{M}{\underset{︸}{I_{\text{p}a2}^{j\text{f}*}}},\underset{K}{\underset{︸}{S_{\text{m}a22}^{k\text{fp}*}}},\underset{K}{\underset{︸}{I_{\text{m}a22}^{k\text{fp}*}}},S_{\text{h}22}^{\text{fpm}*},I_{\text{h}22}^{\text{fpm}*}\right). \end{array}$$

When the poultry of poultry farms has avian influenza, and the poultry of backyard poultry farm has no avian influenza, we can obtain four cases. In four cases, human is most likely transmitted from the poultry farm to the secondary wholesale market, then to the retail live-poultry market, and finally to humans.

***Case 6*.**

If $\underset{1\leq i\leq L}{\min}\left\{R_{\text{f}0}^{i}\right\}>1$ and *R*_b0_ > 1, system (33) has the positive equilibrium as described next.

If $\underset{1\leq j\leq M}{\min}\left\{R_{\text{p}0}^{j1}\right\}>1$, $\underset{1\leq j\leq M}{\max}\left\{R_{\text{p}0}^{j2}\right\}<1$, $\underset{1\leq k\leq K}{\min}\left\{R_{\text{m}0}^{k1}\right\}>1$, and $\underset{1\leq k\leq K}{\max}\left\{R_{\text{m}0}^{k2}\right\}<1$, system (33) has one positive equilibrium:

(45) $$\begin{array}{c} E^{*}=\left(\underset{L}{\underset{︸}{S_{\text{f}a}^{i*}}},\underset{L}{\underset{︸}{I_{\text{f}a}^{i*}}},S_{\text{b}a}^{*},I_{\text{b}a}^{*},\underset{M}{\underset{︸}{S_{\text{p}a1}^{j\text{fb}*}}},\underset{M}{\underset{︸}{I_{\text{p}a1}^{j\text{fb}*}}},\underset{K}{\underset{︸}{S_{\text{m}a11}^{k\text{fbp}*}}},\underset{K}{\underset{︸}{I_{\text{m}a11}^{k\text{fbp}*}}},S_{\text{h}11}^{\text{fbpm}*},I_{\text{h}11}^{\text{fbpm}*}\right). \end{array}$$

If $\underset{1\leq j\leq M}{\min}\left\{R_{\text{p}0}^{j1}\right\}>1$, $\underset{1\leq j\leq M}{\max}\left\{R_{\text{p}0}^{j2}\right\}<1$, and $\underset{1\leq k\leq K}{\min}\left\{R_{\text{m}0}^{k2}\right\}>1$, system (33) has two positive equilibria:

(46) $$\begin{array}{c} E^{1*}=\left(\underset{L}{\underset{︸}{S_{\text{f}a}^{i*}}},\underset{L}{\underset{︸}{I_{\text{f}a}^{i*}}},S_{\text{b}a}^{*},I_{\text{b}a}^{*},\underset{M}{\underset{︸}{S_{\text{p}a1}^{j\text{fb}*}}},\underset{M}{\underset{︸}{I_{\text{p}a1}^{j\text{fb}*}}},\underset{K}{\underset{︸}{S_{\text{m}a11}^{k\text{fbp}*}}},\underset{K}{\underset{︸}{I_{\text{m}a11}^{k\text{fbp}*}}},S_{\text{h}11}^{\text{fbpm}*},I_{\text{h}11}^{\text{fbpm}*}\right),\\ E^{2*}=\left(\underset{L}{\underset{︸}{S_{\text{f}a}^{i*}}},\underset{L}{\underset{︸}{I_{\text{f}a}^{i*}}},S_{\text{b}a}^{*},I_{\text{b}a}^{*},\underset{M}{\underset{︸}{S_{\text{p}a1}^{j\text{fb}*}}},\underset{M}{\underset{︸}{I_{\text{p}a1}^{j\text{fb}*}}},\underset{K}{\underset{︸}{S_{\text{m}a12}^{k\text{fbp}*}}},\underset{K}{\underset{︸}{I_{\text{m}a12}^{k\text{fbp}*}}},S_{\text{h}12}^{\text{fbpm}*},I_{\text{h}12}^{\text{fbpm}*}\right). \end{array}$$

If $\underset{1\leq j\leq M}{\min}\left\{R_{\text{p}0}^{j2}\right\}>1$, $\underset{1\leq k\leq K}{\max}\left\{R_{\text{m}0}^{k2}\right\}<1$, and $\underset{1\leq k\leq K}{\min}\left\{R_{\text{m}0}^{k1}\right\}>1$, system (33) has two positive equilibria:

(47) $$\begin{array}{c} E^{1*}=\left(\underset{L}{\underset{︸}{S_{\text{f}a}^{i*}}},\underset{L}{\underset{︸}{I_{\text{f}a}^{i*}}},S_{\text{b}a}^{*},I_{\text{b}a}^{*},\underset{M}{\underset{︸}{S_{\text{p}a1}^{j\text{fb}*}}},\underset{M}{\underset{︸}{I_{\text{p}a1}^{j\text{fb}*}}},\underset{K}{\underset{︸}{S_{\text{m}a11}^{k\text{fbp}*}}},\underset{K}{\underset{︸}{I_{\text{m}a11}^{k\text{fbp}*}}},S_{\text{h}11}^{\text{fbpm}*},I_{\text{h}11}^{\text{fbpm}*}\right),\\ E^{2*}=\left(\underset{L}{\underset{︸}{S_{\text{f}a}^{i*}}},\underset{L}{\underset{︸}{I_{\text{f}a}^{i*}}},S_{\text{b}a}^{*},I_{\text{b}a}^{*},\underset{M}{\underset{︸}{S_{\text{p}a2}^{j\text{fb}*}}},\underset{M}{\underset{︸}{I_{\text{p}a2}^{j\text{fb}*}}},\underset{K}{\underset{︸}{S_{\text{m}a21}^{k\text{fbp}*}}},\underset{K}{\underset{︸}{I_{\text{m}a21}^{k\text{fbp}*}}},S_{\text{h}21}^{\text{fbpm}*},I_{\text{h}21}^{\text{fbpm}*}\right). \end{array}$$

If $\underset{1\leq j\leq M}{\min}\left\{R_{\text{p}0}^{j2}\right\}>1$ and $\underset{1\leq k\leq K}{\min}\left\{R_{\text{m}0}^{k2}\right\}>1$, system (33) has four positive equilibria:

(48) $$\begin{array}{c} E^{1*}=\left(\underset{L}{\underset{︸}{S_{\text{f}a}^{i*}}},\underset{L}{\underset{︸}{I_{\text{f}a}^{i*}}},S_{\text{b}a}^{*},I_{\text{b}a}^{*},\underset{M}{\underset{︸}{S_{\text{p}a1}^{j\text{fb}*}}},\underset{M}{\underset{︸}{I_{\text{p}a1}^{j\text{fb}*}}},\underset{K}{\underset{︸}{S_{\text{m}a11}^{k\text{fbp}*}}},\underset{K}{\underset{︸}{I_{\text{m}a11}^{k\text{fbp}*}}},S_{\text{h}11}^{\text{fbpm}*},I_{\text{h}11}^{\text{fbpm}*}\right),\\ E^{2*}=\left(\underset{L}{\underset{︸}{S_{\text{f}a}^{i*}}},\underset{L}{\underset{︸}{I_{\text{f}a}^{i*}}},S_{\text{b}a}^{*},I_{\text{b}a}^{*},\underset{M}{\underset{︸}{S_{\text{p}a1}^{j\text{fb}*}}},\underset{M}{\underset{︸}{I_{\text{p}a1}^{j\text{fb}*}}},\underset{K}{\underset{︸}{S_{\text{m}a12}^{k\text{fbp}*}}},\underset{K}{\underset{︸}{I_{\text{m}a12}^{k\text{fbp}*}}},S_{\text{h}12}^{\text{fbpm}*},I_{\text{h}12}^{\text{fbpm}*}\right),\\ E^{3*}=\left(\underset{L}{\underset{︸}{S_{\text{f}a}^{i*}}},\underset{L}{\underset{︸}{I_{\text{f}a}^{i*}}},S_{\text{b}a}^{*},I_{\text{b}a}^{*},\underset{M}{\underset{︸}{S_{\text{p}a2}^{j\text{fb}*}}},\underset{M}{\underset{︸}{I_{\text{p}a2}^{j\text{fb}*}}},\underset{K}{\underset{︸}{S_{\text{m}a21}^{k\text{fbp}*}}},\underset{K}{\underset{︸}{I_{\text{m}a21}^{k\text{fbp}*}}},S_{\text{h}21}^{\text{fbpm}*},I_{\text{h}21}^{\text{fbpm}*}\right),\\ E^{4*}=\left(\underset{L}{\underset{︸}{S_{\text{f}a}^{i*}}},\underset{L}{\underset{︸}{I_{\text{f}a}^{i*}}},S_{\text{b}a}^{*},I_{\text{b}a}^{*},\underset{M}{\underset{︸}{S_{\text{p}a2}^{j\text{fb}*}}},\underset{M}{\underset{︸}{I_{\text{p}a2}^{j\text{fb}*}}},\underset{K}{\underset{︸}{S_{\text{m}a22}^{k\text{fbp}*}}},\underset{K}{\underset{︸}{I_{\text{m}a22}^{k\text{fbp}*}}},S_{\text{h}22}^{\text{fbpm}*},I_{\text{h}22}^{\text{fbpm}*}\right). \end{array}$$

When the poultry of poultry farms has avian influenza, and the poultry of backyard poultry farm has avian influenza, we can obtain four cases. In four cases, human is most likely transmitted from the poultry farm and backyard poultry farm to the secondary wholesale market, then to the retail live-poultry market, and finally to humans, or direct transmission from backyard poultry to humans.

***Remark 3*.**

If we assume that there is an edge between each node of the upper layer and each node of the next layer, that is, each node of the upper layer transport poultry to each node of the next layer in the network, when $\underset{1\leq i\leq L}{\max}\left\{R_{\text{f}0}^{i}\right\}>1$, $\underset{1\leq j\leq M}{\max}\left\{R_{\text{p}0}^{j}\right\}>1$, or $\underset{1\leq k\leq K}{\max}\left\{R_{\text{m}0}^{k}\right\}>1$, according to the actual situation, it can be calculated and analyzed by a similar method. Hence, we omit them here.

## 5. Numerical Simulations

In this section, we first use *L*=3, *M*=2, and *K*=3 submodel to simulate. The course of the infected human is typically 1–4 weeks, and we assume that it is 2.5 weeks on average. Thus, the recovery rate of the infective human is *γ*_h_=1.6/month. The disease-related death rate of the infected human is *α*_h_=0.37. The disease-induced death rates of poultry are assumed to be *α*_f,p,m_=4 × 10^−5^ and *α*_b_=5 × 10^−4^. We assume that human can survive 70 years, and the poultry can survive 2 months in the farm, 1 week in wholesale market, 1 month in wet market, and 8 months in backyard farm, respectively. These rates also referred to removal due to slaughtering. Hence, these rates referred to removal due to slaughtering and the natural death. We take the parameter values as *d*_h_=1.19 × 10^−3^/month, *d*_f_=0.8/month, *d*_p_=*d*_m_=1/month, and *d*_b_=0.125/month, respectively.

We estimate that the number of susceptible poultry population is between 10^7^ and 10^8^, the number of infective poultry population is between 0 and 1000 in farm, the number of susceptible poultry population is between 10^4^ and 10^5^, the number of infective poultry population is between 0 and 500 in live-poultry wholesale market, the number of susceptible poultry population is between 10^2^ and 10^3^, the number of infective poultry population is between 0 and 100 in wet market, and the number of susceptible human population is between 10^7^ and 10^8^ in the region. So, we choose the initial values as (*S*_f*a*_^1^(0), *I*_f*a*_^1^(0), *S*_f*a*_^2^(0), *I*_f*a*_^2^(0), *S*_f*a*_^3^(0), *I*_f*a*_^3^(0))=(5 × 10^7^, 1000,4.9 × 10^7^, 900,4.5 × 10^7^, 800), (*S*_p*a*_^1^(0), *I*_p*a*_^1^(0), *S*_p*a*_^2^(0), *I*_p*a*_^2^(0))=(7 × 10^4^, 200,5 × 10^4^, 100), (*S*_m*a*_^1^(0), *I*_m*a*_^1^(0), *S*_m*a*_^2^(0), *I*_m*a*_^2^(0), *S*_m*a*_^3^(0), *I*_m*a*_^3^(0))=(10^3^, 50, 10^3^, 50, 10^3^, 50), (*S*_b_(0), *I*_b_(0))=(10^4^, 100), and (*S*_h_(0), *I*_h_(0), *R*_h_(0))=(10^7^, 0,0).

The difficulty in parameter estimations is that there is no scientifically or officially reported data of live-poultry transportation in China. The values of *a*_*ij*_, *b*_*jk*_, *l*_*j*_, and *c*_*k*_ used in simulations may be estimated based on living habits of people of regions, the density of human population, and so on. Now, we assume that the transport rates of the backyard poultry are the same to each node, namely, *l*_*j*_=0.1 and *c*_*k*_=0.1, where *j*=1,2, *k*=1,2,3. Let *a*_11_=0.03, *a*_12_=0.04, *a*_21_=0.03, *a*_22_=0.04, *a*_31_=0.05, and  *a*_32_=0.02 and *b*_11_=0.03, *b*_12_=0.03, *b*_13_=0.04, *b*_21_=0.05, *b*_22_=0.02, and  *b*_23_=0.03. We assumed the replenishment rate to be 2 months which is the mean lifetime of farm poultry. Let *A*_f_^1^=2.5 × 10^7^, *A*_f_^2^=2.45 × 10^7^, *A*_f_^3^=2.25 × 10^7^, *A*_b_=833, and *A*_h_=1000, respectively.

The transmission rates from the infective poultry in *k*th wet market to the susceptible human are *β*_*k*h_=1.18 × 10^−9^, *k*=1,2,3. The transmission rate from the infective poultry in backyard farm to the susceptible human is *β*_h_=1.66 × 10^−8^.

**Figure 4:**
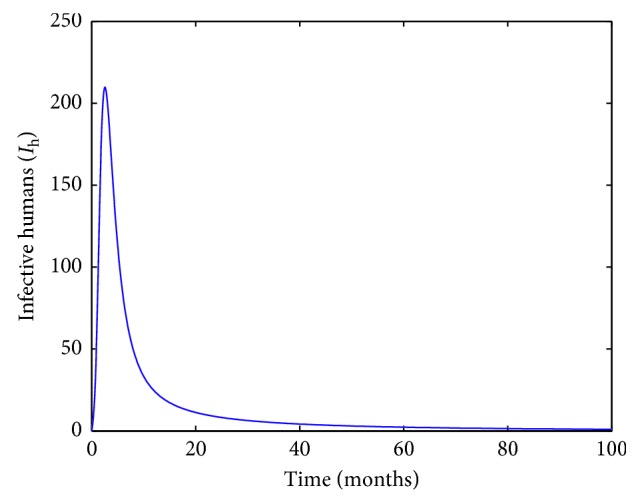
Solution *I*_h_(*t*) is asymptotically stable and converges to the disease-free state value.

The transmission rates from infective poultry to susceptible poultry in different places are *β*_f_=2.78 × 10^−8^, *β*_b_=4.69 × 10^−4^, *β*_p_=2.88 × 10^−8^, and *β*_m_=1.88 × 10^−8^, respectively. Then, *R*_0_=0.9784 < 1. Solution *I*_h_(*t*) is asymptotically stable and converges to the disease-free equilibrium in Figure 4.

**Figure 5:**
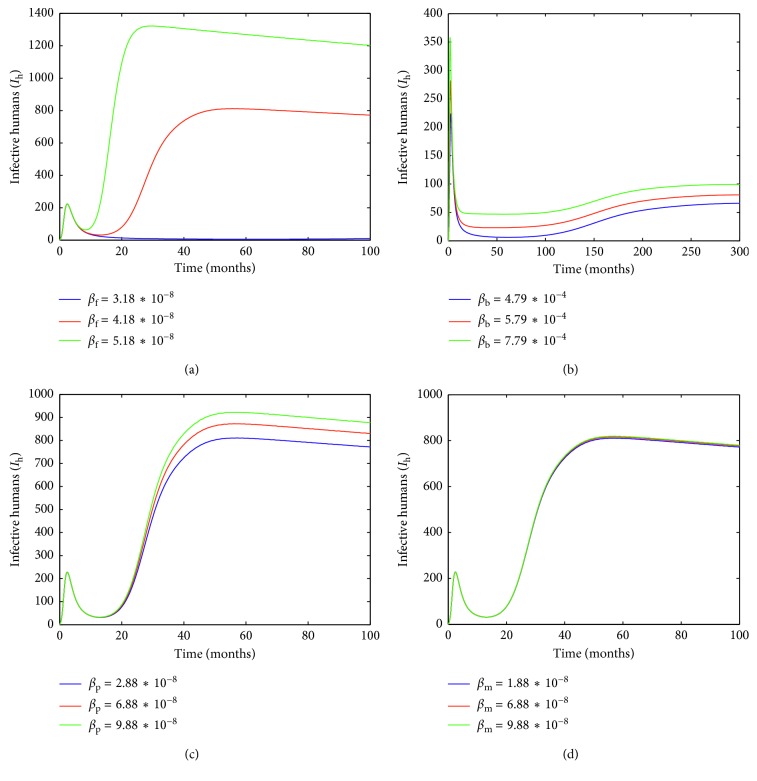
The plots display the changes of *I*_h_(*t*) with *β*_f,b,p,m_ varying.

The transmission rates from infective poultry to susceptible poultry in different places are *β*_b_=4.79 × 10^−4^, *β*_p_=2.88 × 10^−8^, and *β*_m_=1.88 × 10^−8^, respectively. These parameters are fixed. *β*_f_ is varied. Let *β*_f_=3.18 × 10^−8^, *β*_f_=4.18 × 10^−8^, and *β*_f_=5.18 × 10^−8^. From Figure 5, we can see that the beginning is almost the same, but the later is different. Therefore, the transmission rate *β*_f_ has a small impact in the earliest stages but has an important impact on the late disease.

The transmission rates from infective poultry to susceptible poultry in different places are *β*_f_=3.18 × 10^−8^, *β*_p_=2.88 × 10^−8^, and *β*_m_=1.88 × 10^−8^, respectively. These parameters are fixed. *β*_b_ is varied. Let *β*_b_=4.79 × 10^−4^, *β*_b_=5.79 × 10^−4^, and *β*_b_=7.79 × 10^−4^. This only affects the number of infected humans, whereas it has no effect on the arrival time of the peak (Figure 5). Therefore, preventing poultry of backyard poultry farm into the live-poultry market is feasible in a suitable condition.

The transmission rates from infective poultry to susceptible poultry in different places are *β*_f_=3.18 × 10^−8^, *β*_b_=4.79 × 10^−4^, and *β*_m_=1.88 × 10^−8^, respectively. These parameters are fixed. *β*_p_ is varied. Let *β*_p_=2.88 × 10^−8^, *β*_p_=6.88 × 10^−8^, and *β*_p_=9.88 × 10^−8^. The bigger the *β*_p_, the more the human infected (Figure 5). The secondary wholesale market plays an amplifier role.

The transmission rates from infective poultry to susceptible poultry in different places are *β*_f_=3.18 × 10^−8^, *β*_b_=4.79 × 10^−4^, and *β*_p_=2.88 × 10^−8^, respectively. These parameters are fixed. *β*_m_ is varied. Let *β*_m_=1.88 × 10^−8^, *β*_m_=6.88 × 10^−8^, and *β*_m_=9.88 × 10^−8^. The impact is relatively small (Figure 5).

## 6. Conclusion

In this paper, we construct the avian influenza transmission model from poultry (including poultry farm, backyard poultry farm, live-poultry wholesale market, and wet market) to human. We obtain the threshold value for the prevalence of avian influenza and the number of the boundary equilibria and endemic equilibria in different conditions. Numerical simulations show the effects of different transmission rates of different layer on the infected human. And, we can obtain the following cases:

1. The poultry of poultry farm, backyard poultry farm, and poultry wholesale market have no avian influenza, but there is a possible outbreak of avian influenza in wet market (the retail live-poultry market), and avian influenza A (H7N9) virus is most likely transmitted from the retail live-poultry market to humans.
2. The poultry of poultry farm and backyard poultry farm has no avian influenza, but there is a possible outbreak of avian influenza in poultry wholesale market, and then avian influenza A (H7N9) virus is most likely transmitted from the poultry wholesale market to the retail live-poultry market and finally to humans.
3. The poultry of poultry farm has avian influenza, and the poultry of backyard poultry farm has no avian influenza, but there is a possible outbreak of avian influenza in poultry farm, and then avian influenza A (H7N9) virus is most likely transmitted from poultry market to poultry wholesale market, then to the retail live-poultry market, and finally to humans.
4. The poultry of poultry farm has no avian influenza, and the poultry of backyard poultry farm has avian influenza, but there is a possible outbreak of avian influenza in backyard poultry farm, and then avian influenza A (H7N9) virus is most likely transmitted from backyard poultry farm to poultry wholesale market, then to the retail live-poultry market, and finally to humans, or direct transmission from backyard poultry to humans.
5. The poultry of poultry farm and backyard poultry farm has avian influenza, but there is a possible outbreak of avian influenza in poultry farm and backyard poultry farm, and then avian influenza A (H7N9) virus is most likely transmitted from poultry farm and backyard poultry farm to poultry wholesale market, then to the retail live-poultry market, and finally to humans, or direct transmission from backyard poultry to humans.

Hence, the poultry of some nodes on network has avian influenza, and then all edges connected to the node should be cut off. It has a great inhibitory on preventing the spread of disease. So, the network of poultry transportation plays an important role in controlling avian influenza A (H7N9). Moreover, we find that there may have been avian influenza A (H7N9) among humans when there is avian influenza A (H7N9) in the retail live-poultry market, so closing the live-poultry market can reduce the spread of disease to humans at a certain time. In addition, we find that there may have been avian influenza A (H7N9) among humans when there is avian influenza A (H7N9) in the backyard poultry farm. But the spread of backyard poultry to human is quit complex. It can be either direct infection or indirect infection. In China, there are many backyard poultry, so there are still some difficulties in the prevention and control of avian influenza A (H7N9).

## Figures and Tables

**Table 1:** Parameters of system (1).

Parameter	Interpretation
*A*_f_^*i*^	All new recruitments of the avian in *i*th poultry farm
*A*_b_	All new recruitments of the avian in backyard poultry farm
*d*_f,b,p,m_	The natural death rate (including slaughter) of the avian in different places
*α*_f,b,p,m_	The disease-related death rate of the infected avian in different places
*β*_f,b,p,m_	The transmission rate from infective avian to susceptible avian in different places
*a*_*ij*_	The transport rate of individuals from *i*th poultry farm to *j*th live-poultry wholesale market
*b*_*jk*_	The transport rate of individuals from *j*th live-poultry wholesale market to *k*th wet market
*l*_*j*_	The transport rate of individuals from backyard poultry farm to *j*th live-poultry wholesale market
*c*_*k*_	The transport rate of individuals from backyard poultry farm to *k*th wet market
*A*_h_	All new recruitments of the human
*d*_h_	The natural death rate of the human
*β*_*k*h_	The transmission rate from the infective avian in *k*th wet market to the susceptible human
*β*_h_	The transmission rate from the infective avian in backyard farm to the susceptible human
*α*_h_	The disease-related death rate of the infected human
*γ*_h_	The recovery rate of the infective human

## Data Availability

The data used to support the findings of this study are included within the article.
